# Comparison of H_2_O Adsorption and Dissociation Behaviors on Rutile (110) and Anatase (101) Surfaces Based on ReaxFF Molecular Dynamics Simulation

**DOI:** 10.3390/molecules28196823

**Published:** 2023-09-27

**Authors:** He Zhou, Heng Zhang, Shiling Yuan

**Affiliations:** Key Lab of Colloid and Interface Chemistry, Shandong University, Jinan 250100, China

**Keywords:** water dissociation, hydrogen bond network, TiO_2_, ReaxFF, molecular dynamics simulation

## Abstract

The relationship between structure and reactivity plays a dominant role in water dissociation on the various TiO_2_ crystallines. To observe the adsorption and dissociation behavior of H_2_O, the reaction force field (ReaxFF) is used to investigate the dynamic behavior of H_2_O on rutile (110) and anatase (101) surfaces in an aqueous environment. Simulation results show that there is a direct proton transfer between the adsorbed H_2_O (H_2_O_ad_) and the bridging oxygen (O_br_) on the rutile (110) surface. Compared with that on the rutile (110) surface, an indirect proton transfer occurs on the anatase (101) surface along the H-bond network from the second layer of water. This different mechanism of water dissociation is determined by the distance between the 5-fold coordinated Ti (Ti_5c_) and O_br_ of the rutile and anatase TiO_2_ surfaces, resulting in the direct or indirect proton transfer. Additionally, the hydrogen bond (H-bond) network plays a crucial role in the adsorption and dissociation of H_2_O on the TiO_2_ surface. To describe interfacial water structures between TiO_2_ and bulk water, the double-layer model is proposed. The first layer is the dissociated H_2_O on the rutile (110) and anatase (101) surfaces. The second layer forms an ordered water structure adsorbed to the surface O_br_ or terminal OH group through strong hydrogen bonding (H-bonding). Affected by the H-bond network, the H_2_O dissociation on the rutile (110) surface is inhibited but that on the anatase (101) surface is promoted.

## 1. Introduction

TiO_2_ is an important photocatalyst in photocatalytic hydrogen production and photooxidation of organic pollutants [1,2,3]. The interaction of H_2_O with TiO_2_ in an aqueous environment plays an important role in many practical applications [4]. As a fundamental process, water splitting is one of the most important chemical reactions [5]. It affects the reactivity, surface chemistry, and overall performance of the material. For the dissociation of H_2_O by the reaction H_2_O → OH^−^ + H^+^, reactive energy is required and gained from the catalyst or charge transfer [6,7].

Among surfaces relevant to heterogeneous catalysis, rutile and anatase have been extensively investigated for their interaction with water, owing to their photocatalytic activity. To study the adsorption and dissociation characteristics of H_2_O on rutile (110) and anatase (101) surfaces, extensive research using experiments and theoretical calculations has been carried out in the past decades [8]. Many scholars confirmed that H_2_O dissociated at the Ti_5c_ site on the surface of defect-free rutile (110) [9,10]. Using scanning tunneling microscopy (STM), Tan et al. indicated that the dissociative state of H_2_O was more stable than the molecular state of H_2_O at the Ti_5c_ site on the rutile (110) surface [11]. Additionally, the adsorption of H_2_O on the anatase (101) surface was in the molecular form rather than the dissociated form under the vacuum condition [12,13,14].

At the atomic level, it is still a great challenge to characterize the adsorption and dissociation behavior of H_2_O on the TiO_2_ surface under an aqueous environment in experiments. There are still some unsettled fundamental problems and controversies. For example, the nature of adsorbed water, the extent of H_2_O dissociation, and the generation of surface hydroxyl species on the perfect TiO_2_ surface remain controversial. Some experimental measurements and theoretical investigations suggested that molecular adsorption mechanism or mixed adsorption mechanisms may occur simultaneously at room temperature [12,15,16,17,18,19]. Additionally, Ángel et al. directly determined that the terminal hydroxyl group and the bridge hydroxyl group were easily formed on the anatase TiO_2_ surface in an aqueous environment [20,21]. Under this circumstance, even in the well-defined crystal plane, the adsorption and dissociation mechanism of H_2_O on the TiO_2_ surface remains incompletely elucidated [16]. Therefore, it is still necessary to clarify the adsorption and dissociation behavior of H_2_O on the TiO_2_ surface in the real environment.

In the real environment, there is an intermolecular interaction between adsorbed and non-adsorbed H_2_O in bulk water. By the first-principle calculation, double or triple layers of water can be found on TiO_2_ surfaces [22,23]. Additionally, some in situ experiments were also carried out. Using STM measurement, Tan et al. confirmed that the interfacial H-bond facilitated the dehydrogenation of H_2_O on the rutile (110) surface [11]. Yang et al. found that the dissociation of H_2_O on the rutile (110) surface was suppressed at high coverage [24,25]. However, the probability of H_2_O dissociation on the anatase (101) surface remained constant with the increase in H_2_O coverage [26]. An STM experiment showed that the H-bond network on the anatase (001) surface promoted hydrolysis dissociation [27]. However, the role of the H-bond network in H_2_O dissociation on rutile (110) and anatase (101) surfaces has not been completely revealed yet. Hence, it is essential to explore the role of the H-bond network on H_2_O adsorption and dissociation on rutile (110) and anatase (101) surfaces.

As mentioned above, there are still two problems that need to be solved. One is the adsorption and dissociation mechanism of H_2_O on H_2_O–TiO_2_ interfaces in an aqueous environment. The other is that the water structure formed on TiO_2_ surfaces may be affected by the strength of H-bonding. For this purpose, reactive molecular dynamics (RMD) with ReaxFF is employed to study the atomic and molecular behavior of H_2_O on H_2_O–TiO_2_ heterogeneous interaction in an aqueous environment. Here, we demonstrate the significance of subtle surface structures in water dissociation on rutile and anatase TiO_2_ in aqueous environments. This study will offer strategies to achieve efficient catalysis via matching proper surface structures with targeted reaction characteristics. Additionally, the double-layer model is proposed to describe the water structure on rutile (110) and anatase (101) surfaces theoretically. The result shows that the two problems mentioned above are illustrated clearly. 

The rest of the paper is organized as follows. Section 3 introduces the main methods such as the ReaxFF force field. The results and discussion are given in Section 2. The conclusions are summarized in Section 4.

## 2. Results and Discussion

### 2.1. Adsorption and Dissociation Mechanism of H_2_O on Rutile (110)

**Figure 1:**
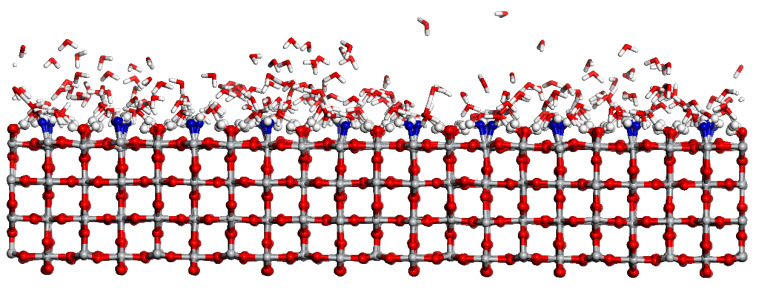
The RMD simulation snapshot of water on rutile (110) at the coverage of 2.0 ML. The blue ball represents the O atom from the adsorbed and dissociated H_2_O.

**Figure 2:**
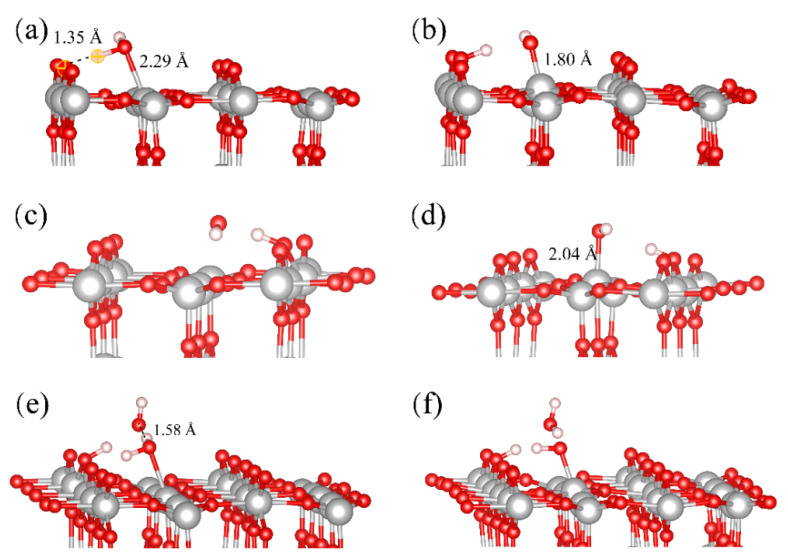
Snapshots of H_2_O adsorption and dissociation on the rutile (110) surface: (**a**,**b**) represent direct dissociation of the H_2_O_ad_ molecule at the Ti_5c_ site; (**c**,**d**) represent the dissociation and adsorption process of H_2_O_f_ molecule at the Ti_5c_ site; (**e**,**f**) represent the indirect dissociation of H_2_O_ad_ at the Ti_5c_ site. Red, grey and pink balls represent O, Ti and H atoms, respectively.

Among various rutile surfaces, rutile (110) is the most stable one and it is widely discussed [28]. Figure 1 shows the RMD simulation snapshot of the water distribution with the coverage of 2.0 ML on the rutile (110) surface. Here, the adsorption and dissociation of H_2_O at the Ti_5c_ site are observed. In Figure 1, the blue ball represents the dissociated H_2_O at the Ti_5c_ site. As shown in Figure 2a,c, the result shows that the mixed state containing the molecular and dissociative adsorption of H_2_O on the rutile (110) surface is favorable. For molecular adsorption, the O atom of H_2_O_ad_ forms a coordination bond with the surface Ti_5c_ atom, and the length of the Ti_5c_-O_ad_ bond is 2.29 Å in Figure 2a. The terminal H_2_O_ad_ forms an intermolecular H-bond with the surface O_br_, in which the distance of the H-bond is 1.35 Å. The interfacial H-bond can effectively assist proton transfer and exchange across the surface. Figure 2b shows that the generated OH group (OH_ad_) is stably adsorbed at the Ti_5c_ site, where the length of the Ti_5c_-O bond is 1.80 Å. This result is consistent with other research using STM experiments and DFT calculations [11,29]. For dissociative adsorption, the terminal free H_2_O (H_2_O_f_) can easily combine with the surface O_br_, resulting in a free OH group (OH_f_) and an O_br_H, as shown in Figure 2c,d. The formation of O_br_H promotes the dissociation of H_2_O_f_. Subsequently, the generated OH_f_ group is stably adsorbed at the adjacent Ti_5c_ site with the length of the Ti_5c_-O bond being 2.04 Å. Alternatively, the OH_f_ group recombines with the O_br_H. Additionally, the indirect dissociation mechanism is also observed on the rutile (110) surface. As shown in Figure 2e,f, the H_2_O in the second layer donates an H-bond to an adjacent O_br_ and transfers its proton to the O_br_. Simultaneously, the H_2_O in the second layer receives a proton from the H_2_O_ad_ at the Ti_5c_ site. This is consistent with other results of DFT studies [30]. However, this indirect proton transfer involves at least two proton transfers, whose sequential occurrence is less likely than the direct proton transfer between the H_2_O_ad_ and the surface O_br_.

**Figure 3:**
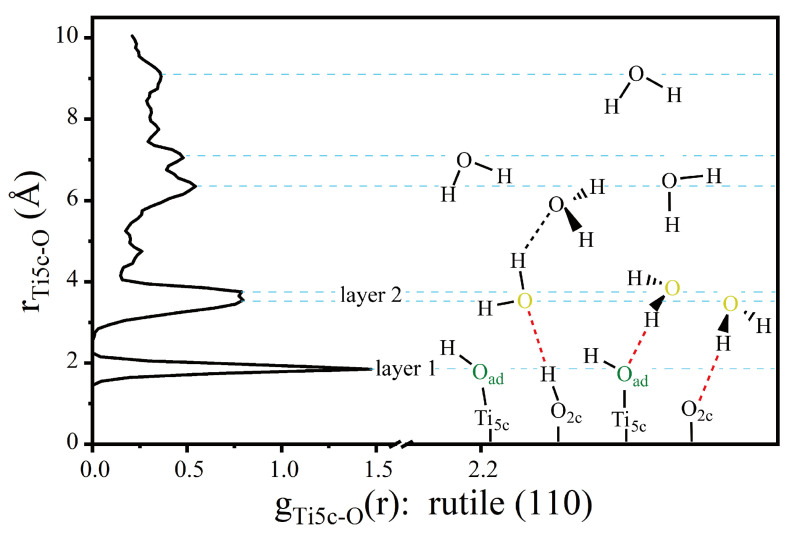
On the left is the RDF of Ti_5c_-O. On the right is the cartoon illustration of the H-bond network on the rutile TiO_2_ (110) surface. The red dotted line represents the enhanced H-bond between the first layer and the second layer of water, and the black dotted line represents the H-bond of ordinary H_2_O-H_2_O.

To manifest the interfacial character more clearly, Figure 3 shows the radial distribution function (RDF) of Ti_5c_-O_w_ (O_w_ represents the O from H_2_O) at the coverage of 3.0 ML. The first peak of r(Ti_5c_-O_w_) is about ~1.85 Å, which corresponds to terminal hydroxyl groups (OH_ad_) from dissociative H_2_O molecules on the rutile (110) surface. The second peaks of r(Ti_5c_-O_w_) appear at about ~3.55 Å and ~3.75 Å. They correspond to the H_2_O in the second layer connected with the surface O_br_ and terminal OH through the H-bond network. The water above the second layer can be regarded as bulk water. These results are consistent with the simulation data obtained by Předota et al. [31]. They observed the first layer of oxygen from the terminal OH group at the top of Ti_5c_ sites with a distance of ~1.9–2.4 Å, and the second layer of water appears at about ~3.8 Å. The double-layered structure of water is also obtained by Mamontov and co-workers [32,33]. As illustrated on the right of Figure 3, the dashed red line represents the H-bond network between the second layer of water and surface O_br_ or OH groups, which affects water dissociation.

**Figure 4:**
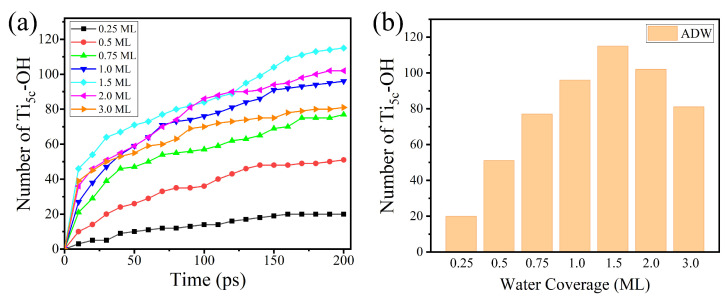
(**a**) The amount of water dissociation varies with time under the different coverage. (**b**) The AWD changes with the coverage of water on the rutile (110) surface.

To investigate the effects of the H-bond network for H_2_O dissociation on the rutile (110) surface, the RMD simulation under different initial coverage is shown in Figure 4a,b. The result shows that the amount of water dissociation (AWD) reaches a maximum value at 1.5 ML coverage with the increase in water coverage. The result is consistent with the previous ab initio MD simulation by Bandura et al. [34]. The possible reason is that the H-bond network formed by the second layer of water inhibits the direct dissociation of H_2_O to a certain extent. At low coverage (<1.5 ML), there is a direct proton transfer between the terminal H_2_O_ad_ molecule and a nearby surface O_br_. At higher coverage (>1.5 ML), H_2_O in the second layer shares the H-bonding with the surface O_br_ and OH group. The direct dissociation of H_2_O_ad_ is inhibited because the surface O_br_ is occupied by the H_2_O in the second layer. However, the possibility of H_2_O_ad_ dissociation via the indirect proton transfer is relatively low. This demonstrates that the dissociation of H_2_O will be suppressed at high coverage on the rutile (110) surface. These results confirm the observation results of the STM experiment by Yang et al. [24]. They suggested that the reaction of H_2_O dissociation was strongly suppressed as the coverage of water increases on the rutile (110) surface. Through the statistics and ensemble averaging of these microscopic processes, our RMD results predict macroscopic properties well.

### 2.2. Adsorption and Dissociation Mechanism of H_2_O on Anatase (101)

**Figure 5:**
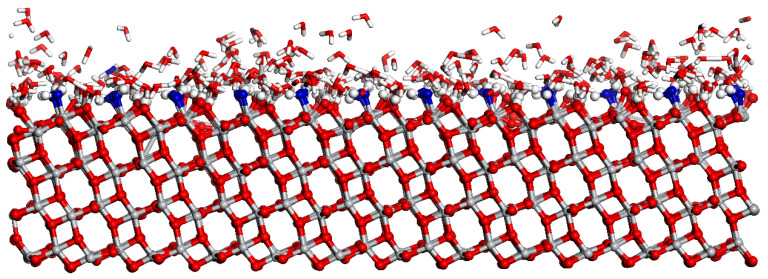
The RMD simulation snapshot of water distribution on the anatase (101) surface at the coverage of 2.0 ML. The blue ball represents the O atom from the adsorbed H_2_O.

**Figure 6:**
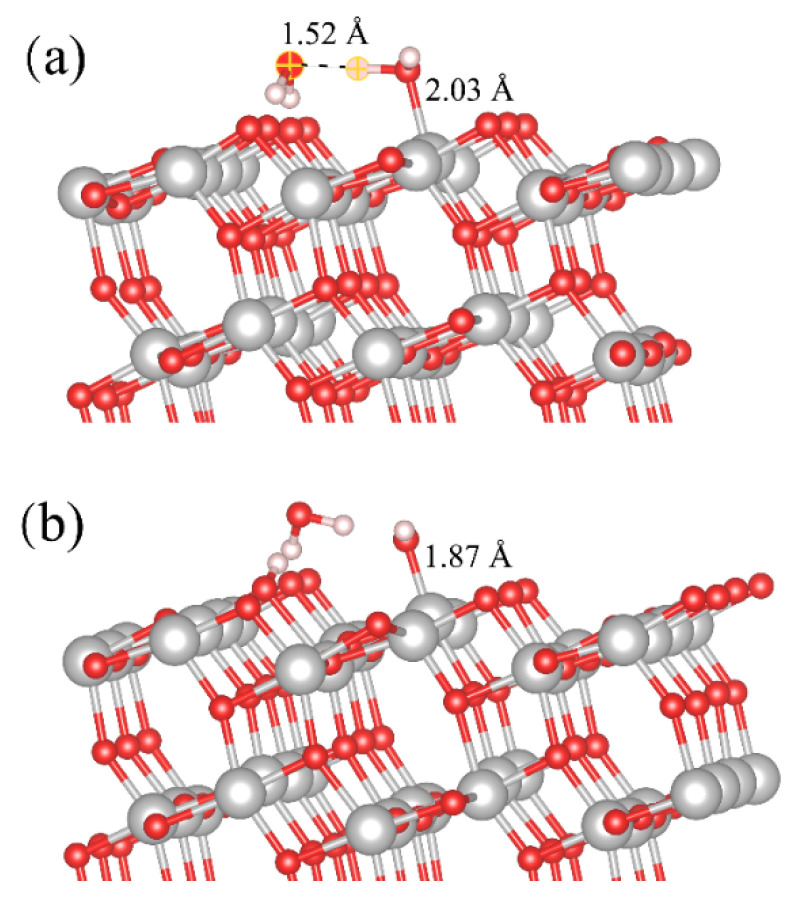
Snapshots of H_2_O adsorption and dissociation on the anatase (101) surface: (**a**) the molecular adsorption of H_2_O_ad_ at the Ti_5c_ site, and (**b**) the indirect dissociation of H_2_O_ad_ at the Ti_5c_ site.

**Figure 7:**
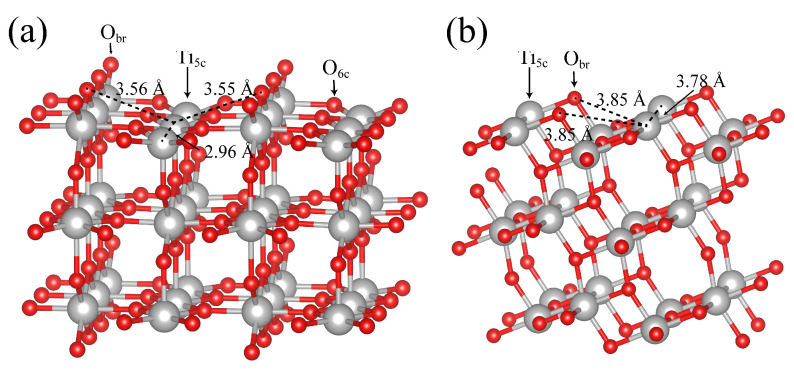
The surface structures of (**a**) rutile (110) and (**b**) anatase (101).

Anatase (101) is the lowest-energy surface of the anatase TiO_2_ polymorph [35]. Figure 5 shows the equilibrium trajectory snapshot of the water distribution over anatase (101) at the coverage of 2.0 ML, in which the blue ball represents the dissociated H_2_O at the Ti_5c_ site. As shown in Figure 6a, the simulation result shows that molecular adsorption is favorable on the anatase (101) surface, which matches with the observation in the experimental result [12]. For molecular adsorption, the O atom of H_2_O_ad_ forms a coordination bond with the surface Ti_5c_ atom, and the length of Ti_5c_-O_ad_ is 2.03 Å in Figure 6a. The H_2_O in the second layer shares the H-bond with both the H_2_O_ad_ and surface O_br_, and forms a water layer in close contact with bulk water. The simulated snapshot in Figure 6b describes the dissociation progress of H_2_O_ad_ on the anatase (101) surface. The H_2_O in the second layer provides a cascaded channel for the proton transfer from the H_2_O_ad_ to the surface O_br_. The RMD simulation result is consistent with the DFT calculation by Selloni et al. [18]. However, during the reaction on the anatase (101) surface, no direct proton transfer is observed between the terminal H_2_O_ad_ molecule and a nearby surface O_br_. This behavior is contrary to that of the rutile (110) surface investigated in this paper. As shown in Figure 6a and Figure S3, the H-bond between the H_2_O_ad_ and the H_2_O in the second layer is 1.52 Å, and that between H_2_O_ad_ and the nearby O_br_ is 2.50 Å. The larger distance between H_2_O_ad_ and the O_br_ site makes the direct proton transfer unfavorable on the anatase (101) surface, which is consistent with the calculation result by others [18,36]. The varied behavior of the water dissociation is found to be related to the subtle structure difference of surface Ti_5c_ and O_br_ sites on rutile (110) and anatase (101). As shown in Figure 7a,b, the rutile (110) surface is flat with O_br_ or O_br_^2−^ bound to 6-fold-coordinated Ti cations and is projected out of the surface plane, whereas the anatase (101) surface has a terraced structure and exposes O_br_ or O_br_^2−^ at the step edge. The distance between Ti_5c_ and O_br_ is about 3.55–3.56 Å on the rutile (110) surface, while that distance is about 3.85 Å on the anatase (101) surface. The larger distance between Ti_5c_ and O_br_ makes the transfer of H atom from H_2_O_ad_ to O_br_ difficult on the anatase (101) surface. Therefore, for the dissociation of H_2_O_ad_ on the anatase (101) surface, it is necessary to assist proton transfer through the H-bond network. 

**Figure 8:**
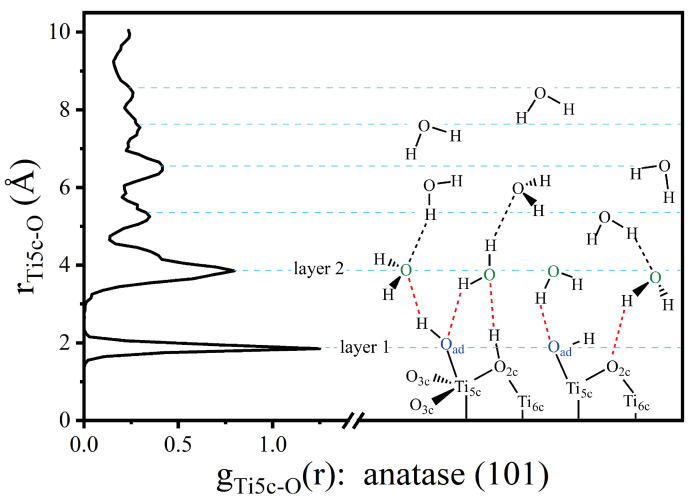
On the left is the RDF of Ti_5c_-O. On the right is the cartoon illustration of the H-bond network on the anatase TiO_2_ (101) surface. The red dotted line represents the enhanced H-bond between the first layer and the second layer of water, and the black dotted line represents the H-bond of ordinary H_2_O-H_2_O.

In order to illustrate interfacial characteristics more clearly, Figure 8 shows the RDF of Ti_5c_-O_w_ on the anatase (101) surface at the coverage of 3.0 ML. The first peak of r(Ti_5c_-O_w_) is estimated at ~1.85 Å, corresponding to the terminal OH_ad_ group. The second peak of r(Ti_5c_-O_w_) appears at about ~3.85 Å, which corresponds to the second layer of H_2_O. The H_2_O in the second layer is connected to the surface O_br_ and terminal OH group through the H-bond network. As shown in Figure 8, the dashed red line represents the H-bond network between the first layer and the second layer of water. The water above the second layer can be regarded as bulk water. The double-layer model explains the experimental and theoretical results well [37,38,39]. These results are consistent with the simulation data obtained by Sumita and co-workers [39]. They showed that the O atom in the first layer was consistent with dissociated H_2_O_ad_ at the Ti_5c_ site, and the RDF of the first peak was at ~1.82 Å by DFT. 

**Figure 9:**
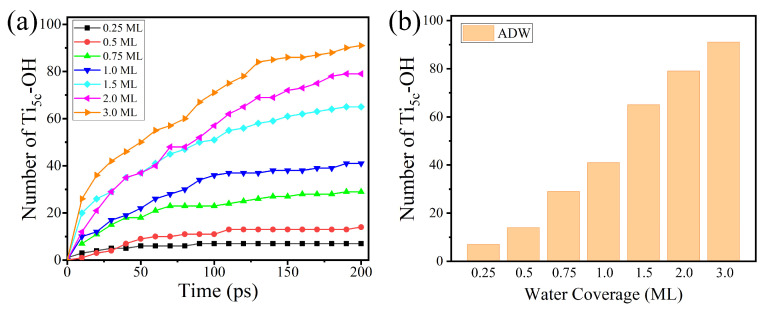
(**a**) The amount of water dissociation varies with time under the different coverage. (**b**) The AWD changes with the coverage of water on the anatase (101) surface.

To investigate the effect of the H-bond network for H_2_O dissociation on the anatase (101) surface, Figure 9a,b exhibit the change of the AWD with different initial coverage in the RMD simulation. However, it is worth noting that the AWD monotonically increases with the increase in coverage. The result suggests that the H_2_O in the second layer participates in and assists the dissociation of H_2_O_ad_. This phenomenon is contrary to the results of the rutile (110) surface studied in this paper. The main reason is that the dissociation of H_2_O happens in different ways on rutile (110) and anatase (101) surfaces. The dissociation of H_2_O on rutile (110) is mainly driven by the proton transfer directly to the surface O_br_, and the H-bond network between the first layer of water and the second layer of water may greatly reduce the dissociation of H_2_O, while the indirect proton transfer on the anatase (101) surface needs to be assisted by the H_2_O in the second layer.

### 2.3. The Roles of the H-Bond Network in Water Dissociation

**Figure 10:**
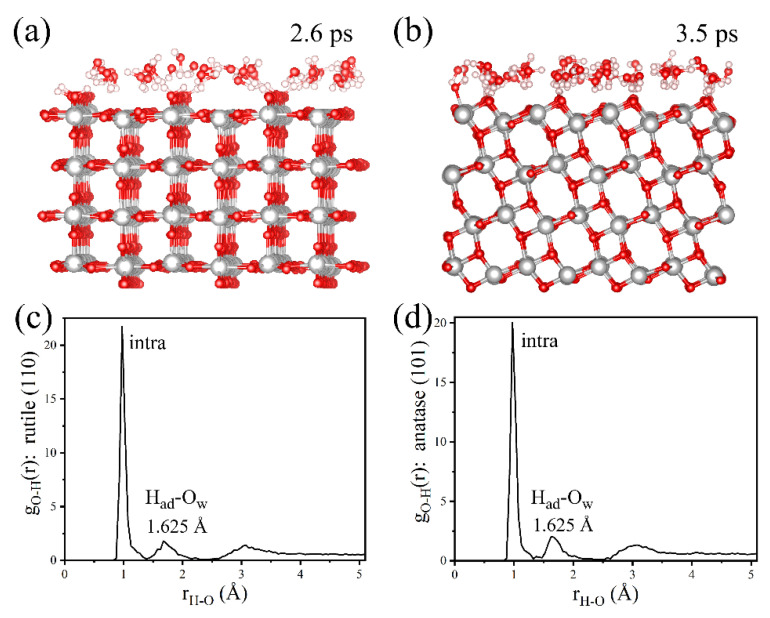
(**a**) A simulated snapshot of local water distribution at 2.6 ps on the rutile (110) surface with the coverage of 3.0 ML; (**b**) a simulated snapshot of local water distribution at 3.5 ps on the anatase (101) surface with the coverage of 3.0 ML; (**c**,**d**) RDFs of O-H on rutile (110) and anatase (101) surfaces, respectively.

**Figure 11:**
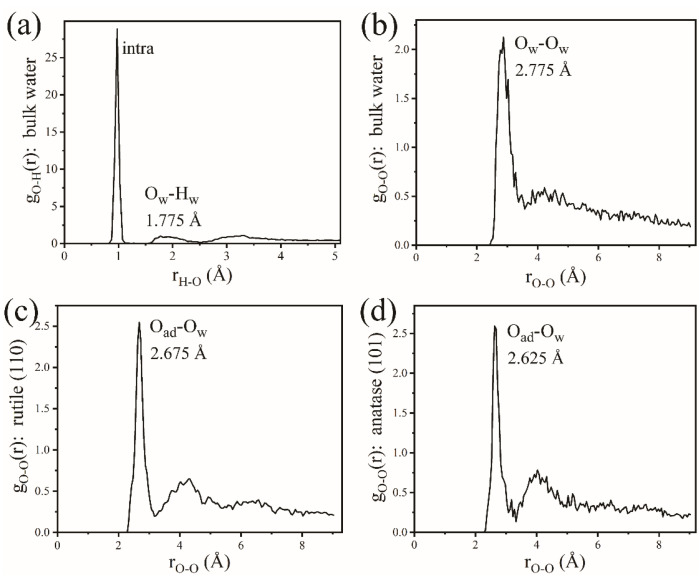
RDFs of (**a**) O_w_-H_w_ in bulk water; (**b**) O_w_-O_w_ in bulk water; (**c**) O-O_w_ on the rutile (110) surface; and (**d**) O-O_w_ on the anatase (101) surface.

As discussed above, the H-bond network, which is ubiquitous in a practical aqueous environment, plays a crucial role in the dissociation of H_2_O on rutile (110) and anatase (101) surfaces. This paper suggests the double-layer model on rutile (110) and anatase (101) surfaces: the first layer is the dissociated H_2_O_ad_ at the Ti_5c_ site, and the second layer is defined as the H_2_O adsorbed onto the O_br_ or terminal OH group through H-bonding. Simulated snapshots in Figure 10a,b show the ordered H-bond network geometry of the second layer of water on the rutile (110) surface at 2.6 ps and the anatase (101) surface at 3.5 ps, respectively. It is observed that the H_2_O in the second layer adsorbed on O_br_ through strong H-bonding interaction. To further understand the effect of strong H-bonding in the dissociation of H_2_O on TiO_2_ surfaces, this paper goes on to investigate the property of the H-bond network in the second layer of water. Figure 10c,d and Figure 11a show the RDFs of O-H on the rutile (110) surface, anatase (101) surface, and bulk water, respectively. The first peak represents the distance of the intramolecular O-H bond. The second peak of r(O-H) corresponds to the strong H-bonding between the H_2_O in the second layer and the surface O_br_ or terminal OH group. For convenience, the second peak of r(O-H) is labeled as r_2_(O_ad_-H_w_). As shown in Figure 10c,d, the r_2_(O_ad_-H_w_) of rutile (110) and anatase (101) is about ~1.63 Å. For comparison, the r_2_(O_ad_-H_w_) in bulk water is estimated at ~1.78 Å in Figure 11a. The shorter r_2_(O_ad_-H_w_) on rutile (110) and anatase (101) surfaces suggests a stronger H-bonding interaction between the H_2_O in the second layer and the surface O_br_ or terminal OH group.

Figure 11b–d, respectively, calculate the RDFs of O-O on the rutile (110) surface, anatase (101) surface, and bulk water. The first peak of r(O-O_w_) represents the distance from the surface O_br_ or O_ad_ to the H_2_O in the second layer on rutile (110) and anatase (101) surfaces. As shown in Figure 11b, the first peak of r(O_w_-O_w_) is located at about ~2.78 Å in the bulk water, which is in accord with the experimental measurement and DFT calculations [40,41]. Compared with that in the bulk water, the first peak of r(O-O_w_) on rutile (110) is estimated at ~2.68 Å in Figure 11c, and that on anatase (101) is about ~2.63 Å in Figure 11d. The shorter r(O-O_w_) indicates the strong interaction between the H_2_O in the second layer and the surface O_br_ or OH group on rutile (110) and anatase (101) surfaces. Therefore, the H-bonding between the surface O_br_ or terminal OH group and the second layer of water appears to be stronger than the H-bonding of ordinary H_2_O-H_2_O in bulk water. 

**Figure 12:**
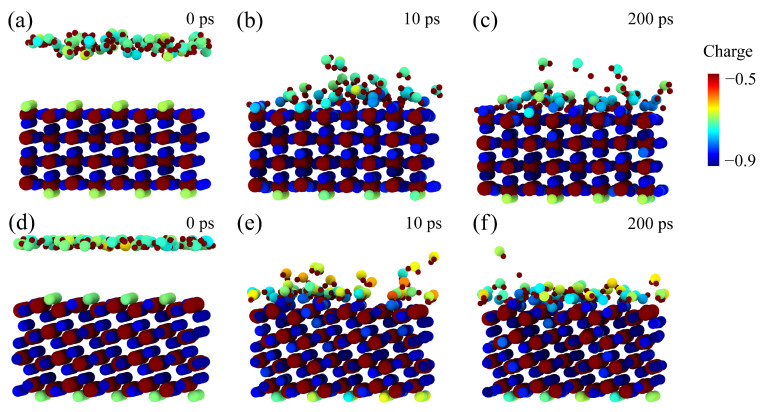
Snapshot views of the local structures of the rutile (110) surface: (**a**) before the interaction at 0 ps, (**b**) after the reaction occurs at 10 ps, and (**c**) after the reaction occurs at 200 ps with the coverage of 1.5 ML. Snapshot views of the local structures of the anatase (101) surface: (**d**) before the interaction at 0 ps, (**e**) after the reaction occurs at 10 ps, and (**f**) after the reaction occurs at 200 ps with the coverage of 1.5 ML. The atom is colored by charge. Green, yellow and blue balls represent O atoms. Smaller and bigger brown balls represent H and Ti atoms, respectively.

Figure 12 shows the change of surface O_br_ and O_ad_ atomic charge during the reaction process. As illustrated in Figure 12a,d, the mean charge of surface O_br_ atoms is close to −0.65 e on the rutile (110) surface, and that on the anatase (101) surface is about −0.70 e before the dissociation reaction of H_2_O. As shown in Figure 12b,c,e,f, the mean charge of O_br_ and O_ad_ atoms dramatically decreases until the reaction is completed. The charge of surface O_br_ and O_ad_ atoms is roughly stable after the reaction. The mean charge of O_br_ and O_ad_ on rutile (110) is −0.77 e and that on anatase (101) is about −0.80 e. In Figure 12c,f, surface O_br_ and O_ad_ atoms are more electron-rich than O_w_ in bulk water (−0.71 e). Correspondingly, the polarization of surface O_br_ and O_ad_ is enhanced. It is indicated that the strong H-bonding between the surface O_br_ or O_ad_ group and the second layer of water is formed. These results provide insights to reveal the role of the H-bond network for water dissociation on rutile (110) and anatase (101) surfaces. More specifically, the H-bond network inhibits the dissociation of H_2_O at high coverage on the rutile (110) surface. But the H-bond network may facilitate the dissociation of H_2_O by linking the proton transfer channel on the anatase (101) surface. 

## 3. Methods

In this paper, molecular dynamic simulation with ReaxFF force field [42,43] is employed to investigate the behavior of H_2_O on rutile (110) and anatase (101) surfaces. The force field parameters are determined from quantum mechanics (QM) based on training sets and experimental results, which can ensure the accuracy of the RMD simulations. The ReaxFF method developed by van Duin uses the bond order relation obtained from the interatomic distance [43,44], which is updated at each RMD or energy minimization step. It allows continuous bond dissociation for all orders at the same time. Therefore, the ReaxFF can be used to describe chemical reactions, including bond formation and bond breaking [42]. 

In the ReaxFF reactive force field, the total (system) energy is given by [45]

(1) $$E_{\mathrm{system}}=E_{\mathrm{bond}}+E_{\mathrm{over}}+E_{\mathrm{under}}+E_{\mathrm{lp}}+E_{\mathrm{val}}+E_{\mathrm{vdWaals}}+E_{\mathrm{coulomb}}$$

The terms in Equation (1) include bond energies (*E_bond_*), the energy to penalize over-coordination of atoms (*E_over_*), the energy to stabilize under-coordination of atoms (*E_under_*), lone-pair energies (*E_lp_*), valence-angle energies (*E_val_*), van der Waals interactions (*E_vdwaals_*) and terms to handle nonbonded Coulomb (*E_coulomb_*), respectively.

This paper employs the Ti/O/H ReaxFF interatomic potential, which is developed by Kim et al. [45]. The force field is carefully used and validated in previous research on the H_2_O–TiO_2_ interface [46,47,48,49]. Using the large-scale atomic/molecular massively parallel simulator (LAMMPS) package [50,51], the RMD calculation with ReaxFF is capable of simulating systems larger than 10^6^ atoms in nanosecond time scales. 

**Figure 13:**
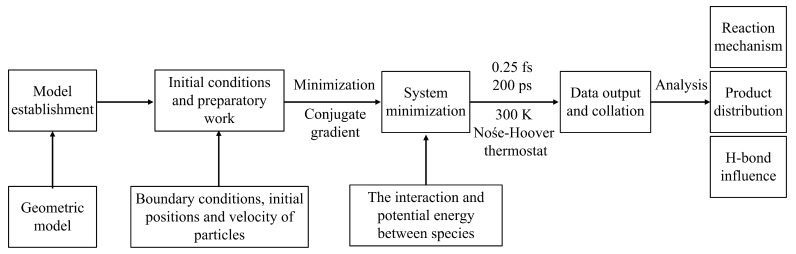
Schematic diagram of the calculation step.

The general flow chart of the molecular dynamics simulation is shown in Figure 13. Rutile (110) and anatase (101) surfaces are carved from bulk rutile and anatase TiO_2_ crystals, respectively. The dimensions of simulation cells are 59.18 Å (x) × 64.97 Å (y) and 67.96 Å (x) × 61.26 Å (y) for rutile (110) and anatase (101) surfaces in Figure S1a,b, respectively. Cleaved rutile (110) and anatase (101) surfaces are composed of four-layer TiO_2_ slabs. Their bottom two layers are fixed in the bulk configuration to simulate the bulk-like environment. There are 200 and 216 Ti_5c_ reactive sites on rutile (110) and anatase (101) surfaces, respectively. In all discussions that follow, one monolayer (ML) is defined as the number of Ti_5c_ sites on rutile (110) and anatase (101) surfaces. H_2_O molecules are placed over rutile (110) and anatase (101) surfaces with a coverage of 0.25, 0.50, 0.75, 1.0, 1.5, 2.0, and 3.0 ML. The simulation box is constructed with periodic boundary conditions in both the X and Y directions. And the fixed boundary condition is applied along the Z direction. To avoid interaction between H_2_O and the bottom of TiO_2_, a reflecting wall is used at the top of the box along the Z direction. The RMD simulation is performed in the canonical ensemble (NVT) with the time step of 0.25 fs. The conjugate gradient (CG) approach is used to minimize energy. During the simulation, the ambient temperature of 300 K is constantly controlled by the Nosé–Hoover thermostat with a 50 fs damping constant [52]. The velocity Verlet algorithm is employed to calculate Newton’s equation of motion. The atomic charge is equilibrated at every time step using the QEq (charge equilibration) model. Ovito is employed to generate snapshots of the simulation. In this paper, all systems reach equilibration after 200 ps, which can be monitored by the convergence of potential energy in Figure S2.

## 4. Conclusions

In this paper, the ReaxFF RMD simulation is employed to investigate the adsorption and dissociation mechanisms of H_2_O on rutile (110) and anatase (101) surfaces in an aqueous environment. Furthermore, this paper explores the vital role of the H-bond network in understanding the underlying mechanisms for water dissociation at a deeper level. Here are several significant findings and conclusions from this paper:

(1) There is a mixed adsorption trend with both molecular and dissociative adsorption on the rutile (110) surface. Compared with that on the rutile (110) surface, molecular adsorption is dominant on the anatase (101) surface.
(2) The dissociation of H_2_O is mainly the direct dissociation on the rutile (110) surface. The interfacial H-bond between the adsorbed H_2_O_ad_ molecule and the surface O_br_ promotes proton transfer for H_2_O dissociation on the rutile (110) surface. Compared with that on the rutile (110) surface, the dissociation of H_2_O is dominated by indirect proton transfer on the anatase (101) surface. This different catalytic function is solely determined by the distance between Ti_5c_ and O_br_ on the surface, which determines the behavior of water dissociation.
(3) The H-bond network plays a crucial role in the dissociation of H_2_O on rutile (110) and anatase (101) surfaces. At high coverage (>1.5 ML), the H-bond network structure of the second layer of water on the rutile (110) surface inhibits the dissociation of H_2_O to some extent. Compared with that on the rutile (110) surface, the RMD simulation shows that H-bond could assist the proton transfer on the anatase (101) surface. In an aqueous environment, the dissociation of H_2_O_ad_ is promoted by the enhanced H-bond network structure of the second layer of water on the anatase (101) surface.

Overall, this paper provides a meaningful insight to understand the behavior of H_2_O adsorption and dissociation on TiO_2_ surfaces in an aqueous environment. It is hoped that the findings reported here will motivate further experimental and theoretical work to achieve a complete understanding of this technologically relevant interface.

## Data Availability

Not applicable.

## Supplementary Materials

The following supporting information can be downloaded at: https://www.mdpi.com/article/10.3390/molecules28196823/s1. Figure S1. Initial models of simulation are established. (a) H_2_O molecules on the rutile (110) surface at the coverage of 2.0 ML. (b) H_2_O molecules on the anatase (101) surface at the coverage of 2.0 ML. Grey, red, and white balls represent Ti, O, and H atoms, respectively. The upper H_2_O is represented by a stick model. Figure S2. The time evolution of potential energy on (a) the rutile (110) surface and (b) the anatase (101) surface during the NVT RMD simulation of water dissociation. Figure S3. The molecular absorption of H_2_O on the anatase (101) surface.
