# Supplementary material for: Comparison of H2O Adsorption and Dissociation Behaviors on Rutile (110) and Anatase (101) Surfaces Based on ReaxFF Molecular Dynamics Simulation
Source: Molecules. 2023 Sep 27;28(19):6823. doi: 10.3390/molecules28196823 (PMC10574456; doi:10.3390/molecules28196823)
Supplement: Supplementary file 1 [file molecules-28-06823-s001.zip › molecules-2609106-supplementary.pdf]

Supporting Information

Comparison of H_2O Adsorption and Dissociation Behaviors on Rutile (110) and Anatase (101) Surfaces Based on ReaxFF Molecular Dynamics Simulation

He Zhou, Heng Zhang, and Shiling Yuan*

1. The calculation models

(a)

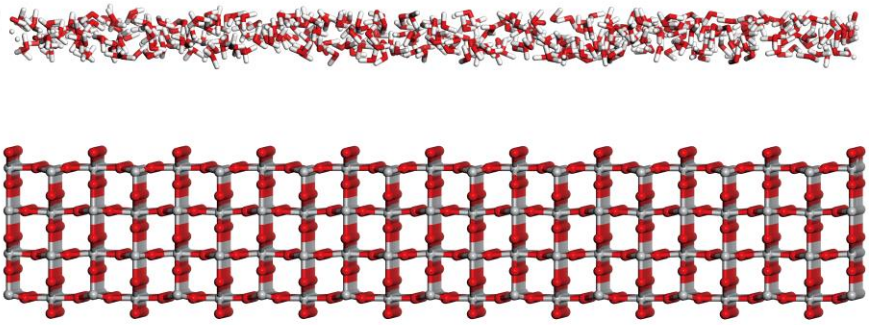

(b)

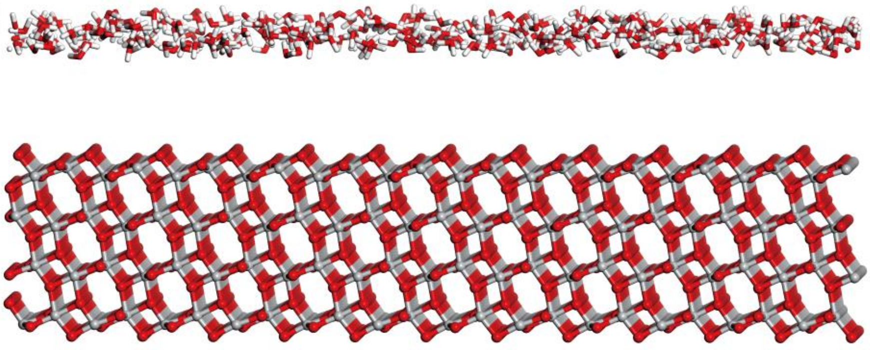

Figure S1. Initial models of simulation are established. (a) H_2O molecules on the rutile (110) surface at the coverage of 2.0 ML. (b) H_2O molecules on the anatase (101) surface at the coverage of 2.0 ML. Grey, red, and white balls represent Ti, O, and H atoms, respectively. The upper H_2O is represented by a stick model.

2. System potential energy

The time evolution of potential energy is calculated. From Figure S1, the result suggests that the system potential energy becomes stable after the water dissociation.

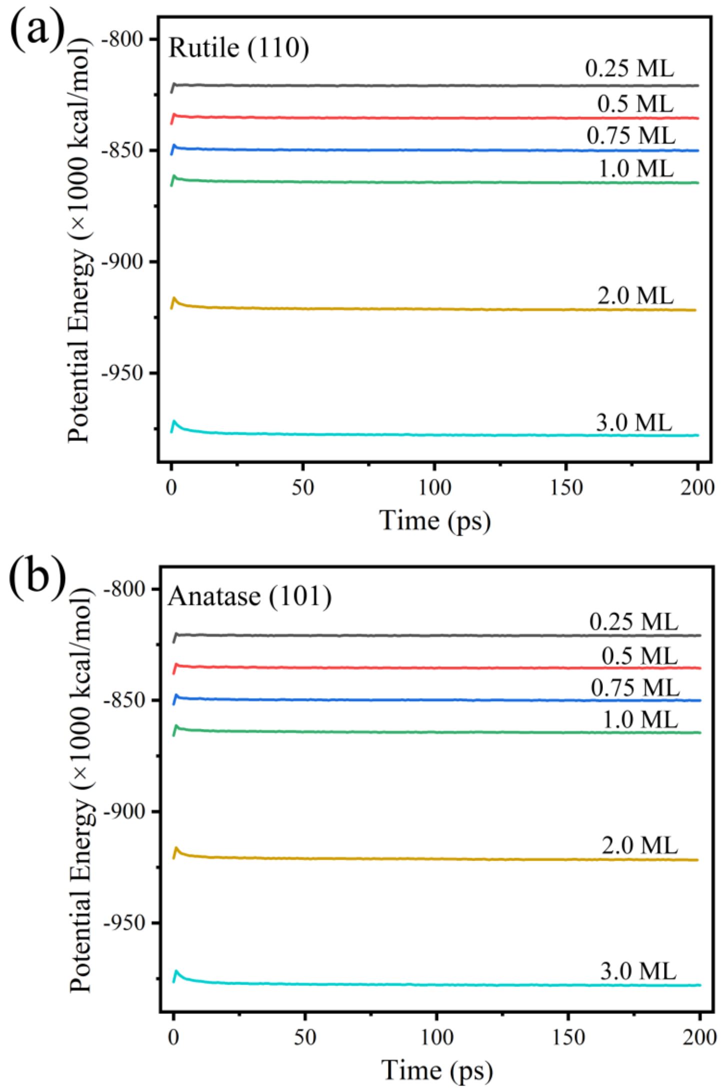

Figure S2. The time evolution of potential energy on (a) the rutile (110) surface and (b) the anatase (101) surface during the NVT RMD simulation of water dissociation.

3. The interfacial H-bond on the anatase (101) surface.

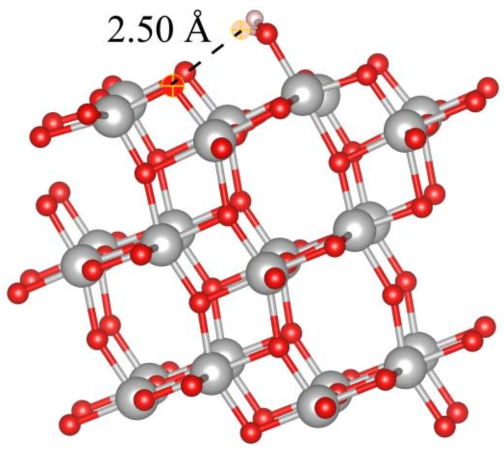

Figure S3. The molecular absorption of H_2O on the anatase (101) surface.
